# Detecting Molecular Features of Spectra Mainly Associated with Structural and Non-Structural Carbohydrates in Co-Products from BioEthanol Production Using DRIFT with Uni- and Multivariate Molecular Spectral Analyses

**DOI:** 10.3390/ijms12031921

**Published:** 2011-03-17

**Authors:** Peiqiang Yu, Daalkhaijav Damiran, Arash Azarfar, Zhiyuan Niu

**Affiliations:** College of Agriculture and Bioresources, University of Saskatchewan, 51 Campus Drive, Saskatoon, SK, S7N 5A8 Canada; E-Mails: dad884@mail.usask.ca (D.D.); ara833@mail.usask.ca (A.A.); zyn847@mail.usask.ca (Z.N.)

**Keywords:** structural and non-structural carbohydrates, co-products from bioethanol processing, molecular spectral analysis

## Abstract

The objective of this study was to use DRIFT spectroscopy with uni- and multivariate molecular spectral analyses as a novel approach to detect molecular features of spectra mainly associated with carbohydrate in the co-products (wheat DDGS, corn DDGS, blend DDGS) from bioethanol processing in comparison with original feedstock (wheat (*Triticum*), corn (*Zea mays*)). The carbohydrates related molecular spectral bands included: A_Cell (structural carbohydrates, peaks area region and baseline: *ca.* 1485–1188 cm^−1^), A_1240 (structural carbohydrates, peak area centered at *ca.* 1240 cm^−1^ with region and baseline: *ca.* 1292–1198 cm^−1^), A_CHO (total carbohydrates, peaks region and baseline: *ca.* 1187–950 cm^−1^), A_928 (non-structural carbohydrates, peak area centered at *ca.* 928 cm^−1^ with region and baseline: *ca.* 952–910 cm^−1^), A_860 (non-structural carbohydrates, peak area centered at *ca.* 860 cm^−1^ with region and baseline: *ca.* 880–827 cm^−1^), H_1415 (structural carbohydrate, peak height centered at *ca.* 1415 cm^−1^ with baseline: *ca.* 1485–1188 cm^−1^), H_1370 (structural carbohydrate, peak height at *ca.* 1370 cm^−1^ with a baseline: *ca.* 1485–1188 cm^−1^). The study shows that the grains had lower spectral intensity (KM Unit) of the cellulosic compounds of A_1240 (8.5 *vs.* 36.6, *P* < 0.05), higher (*P* < 0.05) intensities of the non-structural carbohydrate of A_928 (17.3 *vs.* 2.0) and A_860 (20.7 *vs.* 7.6) than their co-products from bioethanol processing. There were no differences (*P* > 0.05) in the peak area intensities of A_Cell (structural CHO) at 1292–1198 cm^−1^ and A_CHO (total CHO) at 1187–950 cm^−1^ with average molecular infrared intensity KM unit of 226.8 and 508.1, respectively. There were no differences (*P* > 0.05) in the peak height intensities of H_1415 and H_1370 (structural CHOs) with average intensities 1.35 and 1.15, respectively. The multivariate molecular spectral analyses were able to discriminate and classify between the corn and corn DDGS molecular spectra, but not wheat and wheat DDGS. This study indicated that the bioethanol processing changes carbohydrate molecular structural profiles, compared with the original grains. However, the sensitivities of different types of carbohydrates and different grains (corn and wheat) to the processing differ. In general, the bioethanol processing increases the molecular spectral intensities for the structural carbohydrates and decreases the intensities for the non-structural carbohydrates. Further study is needed to quantify carbohydrate related molecular spectral features of the bioethanol co-products in relation to nutrient supply and availability of carbohydrates.

## 1. Introduction

Different types of co-products were produced from bioethanol processing, such as wheat (*triticum*) dried distillers grains with solubles (DDGS), corn (*Zea mays*) DDGS and blend DDGS (e.g., wheat:corn = 70:30). The detailed nutrient profiles of these co-products from bioethanol processing were systematically studied by Nuez-Ortín and Yu [1–4]. In a recent study [5], the effects of bioethanol processing on protein molecular structural changes have been revealed. These changes were significantly associated with metabolizable protein in dairy cattle [6].

To date, none of published studies in literature reported what type of changes occurred in the carbohydrate structures of the co-products after bioethanol processing, compared with original grains.

Carbohydrates include structural carbohydrates such as cellulosic and hemicellulosic compounds or neutral and acid detergent fibers in ruminant nutrition and non-structural carbohydrate such as starch. These carbohydrate structural profiles affect nutrient availability or digestive behavior of the bioethanol co-products.

The objective of this study was to use DRIFT spectroscopy with uni- and multivariate molecular spectral analyses as a novel approach to detect carbohydrate related molecular spectral features of the new co-products from bioethanol processing and quantify carbohydrate related spectral peak intensity for rapid characterization of carbohydrate related molecular structures in the bioethonal bio-products.

## 2. Results and Discussion

### 2.1. Carbohydrate Related Molecular Spectroscopic Features

**Figure 1.**
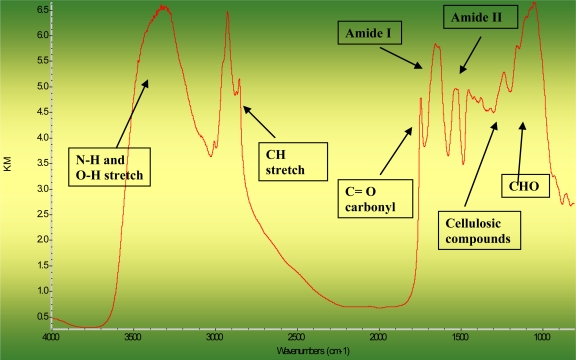
Typical DRIFT molecular spectrum in corn DDGS in the region *ca.* 4000–800 cm^−1^ showed function groups of biopolymers in complex plant system: N-H and O-H stretch, C-H stretch, amide I and II, C=O carbonyl, CHO and cellulosic compounds.

Structural and non-structural carbohydrates profiles affect nutrient availability or digestive behavior. Table 1 shows the structural characteristics of carbohydrates: Comparison between the different grains (wheat, corn) and different types of DDGS (wheat DDGS, corn DDGS and blend DDGS (wheat:corn = 70:30)) from bioethanol production, revealed using the DRIFT mid-infrared molecular spectroscopy. Figure 1 shows a typical DRIFT molecular spectrum in corn DDGS in the region *ca.* 4000–800 cm^−1^ showed function groups of biopolymers in complex plant system: N-H and O-H stretch, C-H stretch, amide I and II, C=O carbonyl, CHO and cellulosic compounds.

Compared wheat with corn, the results shows that there was no significant differences (*P* > 0.05) in the A_Cell (structural CHO, spectral peaks region and baseline: *ca.* 1485–1188) with average 220.1 IR KM units. The A_Cell was associated major hemi- and cellulosic compounds [7,8]. There was no difference (*P* > 0.05) in the A_1240 (structural CHO, peak area centered at *ca.*1240 cm^−1^ with region and baseline: *ca.* 1292–1198 cm^−1^) with average 8.5 IR KM units. The A_1240 spectral parameter is associated with cellulosic compounds [7,8]. There were significant differences (*P* < 0.05) in the A_CHO (total CHO, peaks region and baseline: *ca.* 1187–950 cm^−1^) which is total carbohydrate region [7] and the A_928 (non-structural CHO, spectral peak area centered at *ca.* 928 cm^−1^ with region and baseline: *ca.* 952–910 cm^−1^) which is associated with non-structural carbohydrate. Both the H_1415 (structural CHO, peak height centered at *ca.*1415 cm^−1^ with baseline: *ca.* 1485–1188 cm^−1^) and H_1370 (structural CHO, peak height at *ca.*1370 cm^−1^ with a baseline: *ca.* 1485–1188 cm^−1^) are related to cellulosic compounds [8]. There were no differences (*P* > 0.05) between the wheat and corn (Table 1).

Compared among the co-products (wheat DDGS, corn DDGS and Blend DDGS) (Table 1), there were significant differences (*P* < 0.05) in A_1240 (cellulosic compounds) and A_CHO (total CHO). Both corn DDGS and Blend DDGS were higher (*P* < 0.05) in the A_1240 and A_CHO than wheat DDGS. But there were no differences (*P* > 0.05) between the corn DDGS and blend DDGS. There were no significant differences (*P* > 0.05) in the peak area intensities of the A_Cell (average 241.3), A_928 (average 2.0) and A_860 (average 7.8), and in the peak height intensities of H_1415 (average 1.5) and H_1370 (average 1.2). These results show that molecular spectral profiles differed between the grains and between the co-products. In general, the co-products from bioethanol processing had higher peak intensities in the structural carbohydrates and lower intensities in the non-structural carbohydrates. So far, no published results could be used to compare with the results from this study. The different structural and non-structural carbohydrate molecular spectral profiles may be highly related to carbohydrate functionality and quality.

### 2.2. Detect Changes in Carbohydrate Molecular Structure Changes by Bioethanol Processing

Table 2 shows the comparison between the grains and co-products (DDGS) from bioethanol production in the carbohydrate structural characteristics, revealed using the DRIFT mid-infrared molecular spectroscopy. The results show that the grains had significantly lower peak area intensity of A_1240 (8.5 *vs*. 36.6, *P* < 0.05), higher A_928 (17.3 *vs.* 2.0, *P* < 0.05) and higher A_860 (20.7 *vs.* 7.6, *P* < 0.05) than their co-products from bioethanol processing. There were no differences (*P* > 0.05) in the peak area intensities of A_Cell and A_CHO with average peak area intensities of 226.8 and 508.1 IR KM units, respectively. There were no differences (*P* > 0.05) in peak height intensities of H_1415 and H_1370 with average peak height intensities of 1.35 and 1.15 IR KM unit, respectively. Again no publications were found in this area.

The results indicate that the co-products and grain differed in carbohydrate structure. Bioethanol processing changes original grain carbohydrate molecular structure profiles. It increased structural carbohydrate profiles and decreased non-structural carbohydrate profiles. It is expected that these structural differences may impact the co-products carbohydrate utilization and availability. The results demonstrate that molecular spectral analytical technique may reveal differences in carbohydrate molecular structure.

### 2.3. Discriminate and Classify Carbohydrate Molecular Structure

Infrared spectra based on similarity with other spectra could be clustered using CLA analysis [5]. In this study, the Ward’s algorithm method was used without any prior parameterization of the spectral data in the four different IR regions:

Region 1 is the mid-infrared region *ca.* 4000–827 cm^−1^;
Region 2 is the fingerprint region *ca.* 1800–827 cm^−1^;
Region 3 is the region mainly associated with hemi-and cellulosic carbohydrates [7,8] *ca.* 1452–1188 cm^−1^;
Region 4 is the region mainly total carbohydrate region [7,8] *ca.* 1187–950 cm^−1^.

**Figure 2.**
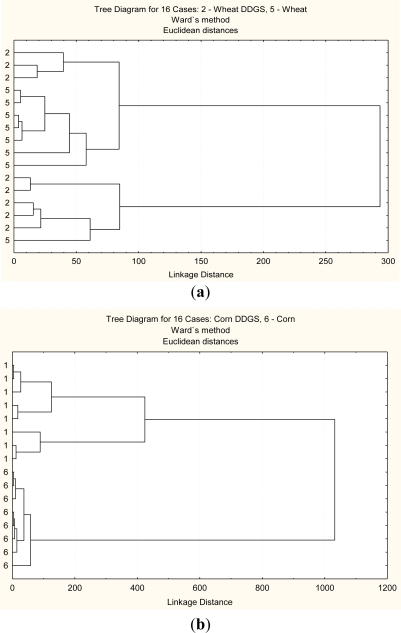
Multivariate molecular spectral analyses of the co-products from bioethanol production at the whole mid-infrared region (4000–827 cm^−1^): CLA cluster analyses of molecular spectrum (Region *ca.* 4000–827 cm^−1^; Distance method: Euclidean; Cluster method: Ward’s algorithm); (**a**) wheat DDGS (code 2) *vs.* wheat (code 5); (**b**) corn DDGS (code 1) *vs.* corn (code 6).

**Figure 3.**
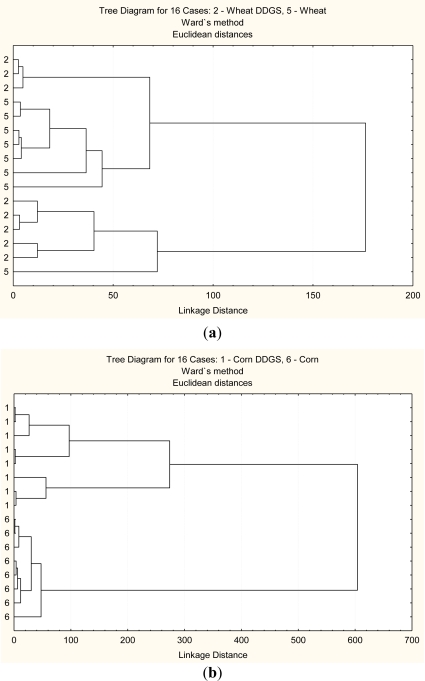

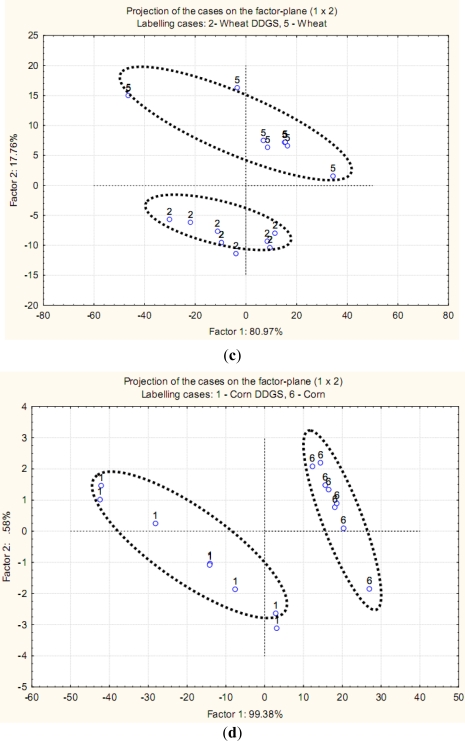
Multivariate molecular spectral analyses of the co-products from bioethanol production at the fingerprint region (*ca.* 1800–827 cm^−1^): CLA cluster analyses of molecular spectrum (Region *ca.* 1800–827 cm^−1^; Distance method: Euclidean; Cluster method: Ward’s algorithm); Principal component analysis (PCA) analyses of molecular mid-IR spectrum. (**a**,**c**) wheat DDGS (code 2) *vs.* wheat (code 5); (**b**,**d**) corn DDGS (code 1) *vs.* corn (code 6). (**a**) Cluster analysis: molecular structure of wheat *vs.* molecular structure of wheat DDGS; (**b**) Cluster analysis: molecular structure of corn *vs*. molecular structure of corn DDGS; (**c**) PCA: molecular structure of wheat *vs*. molecular structure of wheat DDGS. 1st *vs.* 2nd principal component; (**d**) PCA: molecular structure of corn *vs.* molecular structure of corn DDGS. 1st *vs.* 2nd principal component.

**Figure 4.**
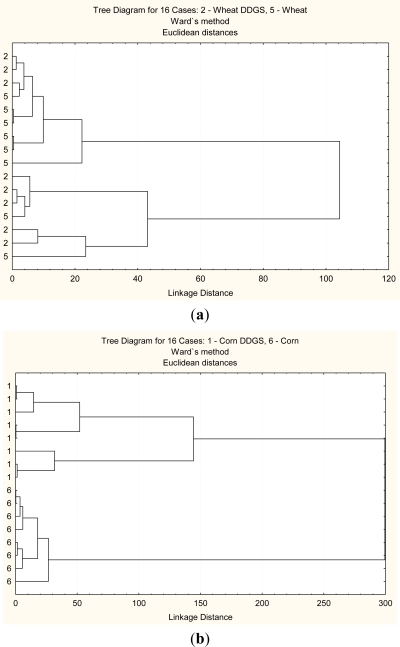

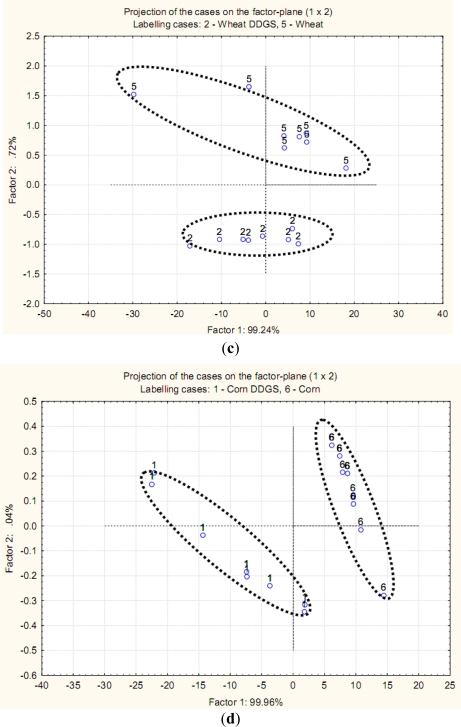
Multivariate molecular spectral analyses of the co-products from bioethanol production at the hemi-and cellulosic compounds region (*ca.* 1452–1188 cm^−1^): CLA cluster analyses of molecular spectrum (Distance method: Euclidean; Cluster method: Ward’s algorithm); Principal component analyses (PCA) of molecular mid-IR spectrum. (**a**,**c**) wheat DDGS (code 2) *vs.* wheat (code 5); (**b**,**d**) corn DDGS (code 1) *vs.* corn (code 6). (**a**) Cluster analysis: molecular structure of wheat vs. molecular structure of wheat DDGS; (**b**) Cluster analysis: molecular structure of corn *vs.* molecular structure of corn DDGS; (**c**) PCA: molecular structure of wheat *vs*. molecular structure of wheat DDGS. 1st *vs.* 2nd principal component; (**d**) PCA: molecular structure of corn *vs.* molecular structure of corn DDGS. 1st *vs.* 2nd principal component.

**Figure 5.**
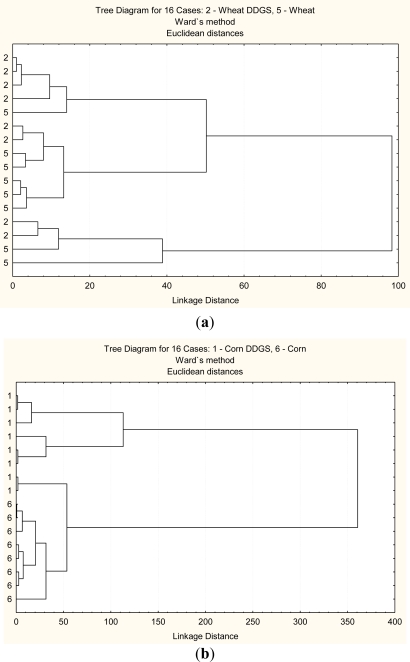

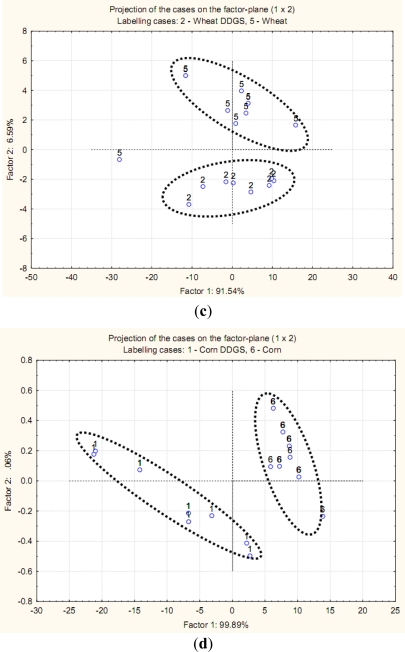
Multivariate molecular spectral analyses of the co-products from bioethanol production at the total carbohydrate region (*ca.* 1187–950 cm^−1^): CLA cluster analyses of molecular spectrum (Distance method: Euclidean; Cluster method: Ward’s algorithm); Principal component analysis (PCA) analyses of molecular mid-IR spectrum. (**a**,**c**) wheat DDGS (code 2) *vs.* wheat (code 5); (**b**,**d**) corn DDGS (code 1) *vs.* corn (code 6). (**a**) Cluster analysis: molecular structure of wheat *vs.* molecular structure of wheat DDGS; (**b**) Cluster analysis: molecular structure of corn *vs.* molecular structure of corn; (**c**) PCA: molecular structure of wheat *vs.* molecular structure of wheat DDGS. 1st *vs.* 2nd principal component; (**d**) PCA: molecular structure of corn *vs.* molecular structure of corn DDGS. 1st *vs.* 2nd principal component.

This method helps discriminate in the structural differences between the grain and its co-products. Figures 2–5 show that two classes can be clear distinguished between corn and corn DDGS, but not between wheat and wheat DDGS. These results indicate that molecular structure between the corn and bioethanol co-product (corn DDGS) were different. These results also indicate that different cereal grains have different responses to bioethanol processing and different sensitivity to heating and fermentation.

The second multivariate analysis tested was a PCA analysis which is a statistical data reduction method [5]. In this study, PCA analysis was used to identify the main sources of variation in carbohydrate conformation at four different regions as mentioned before. Figures 2–5 show that corn and corn DDGS can be grouped in separate ellipses (Figures 2–5d) with no overlapping of groups. The first PC can explain 99% of the variation in the four different spectrum data sets

## 3. Experimental Section

### 3.1. Co-Products from Bioethanol Production

The co-products from bioethanol production—wheat DDGS, blend DDGS (wheat:corn = 70:30), and corn DDGS as well as original feedstock wheat and corn grains were collected. The chemical characteristics, protein and carbohydrate subfraction profiles, energy values [1], rumen and intestinal disappearance from large samples of the DDGS in bioethanol plants [3,4], and modeling nutrient supply were reported [2].

### 3.2. Infrared Spectroscopy

The experiments were carried out at Saskatchewan Structure Sciences Center (SSSC, Saskatoon, Canada). The methodology to prepare samples for molecular spectroscopy study was published before [5]. Each sample from bioethanol production were finely ground (Retsch ZM-1, Brinkmann Instruments (Canada) LTD, Ontario). Diffuse reflectance infrared (KM unit) Fourier transform spectroscopy was performed using a Bio-Rad FTS-40 with a ceramic IR source and MCT detector (Bio-Rad laboratories, Hercules, California, USA). Data was collected using Win-IR software. Spectra were generated from the mid-IR (4000–800 cm^−1^) portion of the electromagnetic spectrum with 256 co-added scans and a spectral resolution of 4 cm^−1^.

### 3.3. Molecular Spectral Analysis of Carbohydrates

Molecular spectral analysis was done with OMNIC 7.2 software (Spectra Tech., USA). The carbohydrate related molecular spectral bands included: A_Cell (structural CHO, peaks area region and baseline: *ca.* 1485–1188 cm^−1^), A_1240 (structural CHO, peak area centered at *ca.* 1240 cm^−1^ with region and baseline: *ca.* 1292–1198 cm^−1^), A_CHO (total CHO, peaks region and baseline: *ca.* 1187–950 cm^−1^), A_928 (non-structural CHO, peak area centered at *ca.* 928 cm^−1^ with region and baseline: *ca.* 952–910 cm^−1^), A_860 (non-structural CHO, peak area centered at *ca.* 860 cm^−1^ with region and baseline: *ca.* 880–827 cm^−1^), H_1415 (structural CHO, peak height centered at *ca.* 1415 cm^−1^ with baseline: *ca.* 1485–1188 cm^−1^), H_1370 (structural CHO, peak height at *ca.* 1370 cm^−1^ with a baseline: *ca.* 1485–1188 cm^−1^). The above carbohydrate associated bands were identified according to the published reports [7–9] and discussed in Yu *et al.* [10].

### 3.4. Statistical Analysis

Statistical analyses were performed using the MIXED procedure of SAS (version 9.1.3). The model used for the analysis was: *Y_ij_* = *μ* + *T_i_* + *e_ij_*, where, *Y_ij_* is an observation of the dependent variable *ij* (molecular spectral parameters: A_Cell, A_1240, A_CHO, A_928, A_860, H_1415, H_1370); *μ* is the population mean for the variable; *T_i_* is the effect of feeds, as a fixed effect, and *e_ij_* was the random error associated with the observation *ij*. To compare grain and bioethanol co-products, the model used for the analysis is: *Y_ij_* = *μ* + *T_i_* + *e_ij_*, where, *Y_ij_* is an observation of the dependent variable *ij* (molecular spectral parameters: A_Cell, A_1240, A_CHO, A_928, A_860, H_1415, H_1370 in IR KM Units); *μ* is the population mean for the variable; *T_i_* is the effect of feed (*i*: 1 = grains; 2 = bioethanol co-products) as a fixed effect, and *e_ij_* is the random error associated with the observation *ij*. The normality check was used Proc Univariate with Normal and Plot options in SAS

For all statistical analyses, significance was declared at *P* < 0.05 and trends at *P* ≤ 0.10. Differences among the treatments were evaluated using a multiple comparison test following the Tukey-Karmer method.

### 3.5. Multivariate Molecular Spectral Analysis for DRIFT Spectra

Multivariate molecular spectral analyses, principal component analysis (PCA) and hierarchical cluster analysis (CLA), were performed using Statistica software 6.0 (StatSoft Inc, Tulsa, OK, USA) to classify and distinguish between the inherent structures.

## 4. Conclusion

In conclusion, the study shows that the co-products and grain differed in carbohydrate structure conformation. Bioethanol processing changed original grain carbohydrate molecular structure. In general, the bioethanol processing increased the molecular spectral intensities for the structural carbohydrates and decreased the intensities for the non-structural carbohydrate. These structural differences may impact the co-products’ carbohydrate utilization and availability. The results demonstrate that molecular spectral analytical technique-DRIFT could be used to reveal differences in carbohydrate molecular structures of grains affected by bioethanol processing. Further study is needed to quantify carbohydrate related molecular spectral features of the bioethanol co-products in relation to nutrient supply and availability of carbohydrates.

## Figures and Tables

**Table 1.** The structural characteristics of carbohydrates: Comparison between different grains (wheat, corn) and different types of DDGS [wheat DDGS, corn DDGS and blend DDGS (wheat:corn = 70:30)] from bioethanol production, revealed using DRIFT mid-infrared molecular spectroscopy.

					**Molecular characteristics (IR KM intensity unit)**					
					**Grain**		**Co-products**			
**Items**	**Peak type**	**Peak center (cm^−1^)**	**Region (cm^−1^)**	**Baseline (cm^−1^)**	**Wheat (*n*= 8)**	**Corn (*n*= 8)**	**Wheat DDGS (*n*= 16)**	**Corn DDGS (*n*= 8)**	**Blend DDGS (*n*= 8)**	***SEM***
						Based on the peak area				
A_Cell	Peak area	–	1485–1188	1485–1188	260.5 ^a b^	179.7 ^b^	210.8 ^a,b^	240.0 ^a,b^	273.0 ^a^	21.14
A_1240	Peak area	*ca.* 1240	1292–1198	1292–1198	10.9 ^c^	6.1 ^c^	30.5 ^b^	44.1 ^a^	41.4 ^a^	2.92
A_CHO	Peak area		1187–950	1187–950	664.4 ^a^	294.6 ^b^	505.8 ^a^	568.5 ^a^	566.6 ^a^	52.30
A_928	Peak area	*ca.* 928	952–910	952–910	19.5 ^a^	15.1 ^b^	2.1 ^c^	1.7 ^c^	2.2 ^c^	0.68
A_860	Peak area	*ca.* 860	880–827	880–827	22.2 ^a^	19.3 ^a^	7.0 ^b^	9.2 ^b^	7.2 ^b^	1.46
						Based on the peak height				
H_1415	Peak height	*ca.* 1415	–	1485–1188	1.5	1.0	1.4	1.5	1.6	0.14
H_1370	Peak height	*ca.* 1370	–	1485–1188	1.4	1.0	1.0	1.2	1.3	0.10

*SEM* = pooled standard error of means; Means with the different letter in the same column are significantly different (*P* < 0.05).

Multi-treatment comparison method: Tukey-Karmer Method.

**Table 2.** The structural characteristics of carbohydrates: Comparison between grains and co-products (DDGS) from bioethanol production, revealed using DRIFT mid-infrared molecular spectroscopy.

		**Molecular Spectroscopy: (IR Peak area intensity KM unit)**					**Molecular Spectroscopy: (IR Peak height intensity KM unit)**	
Items	Replications	A_Cell	A_1240	A_CHO	A_928	A_860	H_1415	H_1370
Peak type		Peaks area	Peak area	Peaks area	Peak area	Peak area	Peak height	Peak height
Peak center (cm^−1^)		–	*ca.* 1240	–	*ca.* 928	*ca.* 860	*ca.* 1415	*ca.* 1370
Region (cm^−1^)		1485–1188	1292–1198	1187–950	952–910	880–827	–	–
Baseline (cm^−1^)		1485–1188	1292–1198	1187–950	952–910	880–827	1485–1188	1485–1188

Grain *vs.* Bioethanol co-products								
Grains	16	220.1	8.5 ^b^	479.5	17.3 ^a^	20.7 ^a^	1.3	1.2
DDGS	32	233.5	36.6 ^a^	536.7	2.0 ^b^	7.6 ^b^	1..4	1.1
*SEM*		14.90	2.16	40.67	0.51	0.83	0.09	0.07
*P* value		0.53	<0.01	0.32	<0.01	<0.01	0.16	0.69

*SEM* = Pooled standard error of means. Means with the same letter in the same column are not significantly different (*P* > 0.05).

Multi-treatment comparison method: Tukey-Karmer Method.
